# RETRACTED: Atiq et al. Vitamin E Analog Trolox Attenuates MPTP-Induced Parkinson’s Disease in Mice, Mitigating Oxidative Stress, Neuroinflammation, and Motor Impairment. *Int. J. Mol. Sci.* 2023, *24*, 9942

**DOI:** 10.3390/ijms27188268

**Published:** 2026-09-17

**Authors:** Abubakar Atiq, Hyeon Jin Lee, Amjad Khan, Min Hwa Kang, Inayat Ur Rehman, Riaz Ahmad, Muhammad Tahir, Jawad Ali, Kyonghwan Choe, Jun Sung Park, Myeong Ok Kim

**Affiliations:** 1 Division of Life Science and Applied Life Science (BK21 FOUR), College of Natural Sciences, Gyeongsang National University, Jinju 52828, Republic of Korealhj4912@gnu.ac.kr (H.J.L.); inayaturrehman201516@gnu.ac.kr (I.U.R.); riazk0499@gnu.ac.kr (R.A.); k.choe@gnu.ac.kr (K.C.);; 2 Department of Psychiatry and Neuropsychology, School for Mental Health and Neuroscience (MHeNs), Maastricht University, 6229ER Maastricht, The Netherlands; 3 Alz-Dementia Korea Co., Jinju 52828, Republic of Korea

The Journal retracts the article “Vitamin E Analog Trolox Attenuates MPTP-Induced Parkinson’s Disease in Mice, Mitigating Oxidative Stress, Neuroinflammation, and Motor Impairment” [1] cited above.

Following publication, concerns were brought to the attention of the Editorial Office relating to the potential duplication of images presented in this article [1] and in two other publications [2,3].

In accordance with the journal’s standard procedure, the Editorial Office and Editorial Board conducted an investigation that identified concerns regarding the validity of the data presented in Figure 1. In addition, duplication was identified between panels presented in Figure 2 [1] and figures published in later articles [2,3], produced by a similar authorship group and presenting different experimental conditions. Although the authors cooperated with the Editorial Office during the investigation, they were unable to provide a satisfactory explanation for the concerns relating to the figures or appropriate raw data for evaluation by the Editorial Board. Consequently, the Editorial Board has lost confidence in the reliability of the findings and has decided to retract this publication [1], as per MDPI’s retraction policy.

This retraction was approved by the Editor-in-Chief of the journal *International Journal of Molecular Sciences*.

The authors have been informed of this retraction but did not provide a comment on this decision.
